# Observation of the curative effect of Guizhi-Shaoyao-Zhimu decoction combined with methotrexate in the treatment of early rheumatoid arthritis based on ultrasonic evaluation: study protocol of a randomized, double-blind, controlled clinical trial

**DOI:** 10.1186/s13063-021-05579-w

**Published:** 2021-11-03

**Authors:** Jiaying Shen, Qianwen Chen, Guanghui Yang, Yikun He

**Affiliations:** 1 Department of Rheumatism, Shuguang Hospital Affiliated to Shanghai University of Traditional Chinese Medicine, Shanghai, 200021 China; 2 Shanghai University of Traditional Chinese Medicine, Shanghai, 201203 China

**Keywords:** Guizhi-Shaoyao-Zhimu decoction, Efficacy, Rheumatoid arthritis, MSUS, Randomized double-blind controlled clinical trial

## Abstract

**Background:**

Rheumatoid arthritis (RA) is a chronic inflammatory autoimmune disease with the primary clinical symptoms of joint swelling and pain. Early detection of erosion and synovial inflammation at an active stage resulting from RA can prevent damage to the joints and activity restriction. However, there are still many patients who do not respond to these treatments; the development of newer, safer drugs is urgently needed. Compared to Western medicine, Guizhi-Shaoyao-Zhimu decoction has equal or higher efficacy and safety for RA patients. With the widespread use of musculoskeletal ultrasound (MSUS), this technology holds great value for the degree of joint damage in RA patients, guiding the clinical selection of treatments, and assessing of prognosis. Therefore, we designed a double-blinded randomized controlled clinical trial to measure the safety and efficacy of Guizhi-Shaoyao-Zhimu decoction in treating early-stage RA using MSUS.

**Methods:**

This study is a randomized, double-blinded, parallel group, placebo-controlled trial. A total of 152 adult participants with early RA will be enrolled, with balanced treatment allocation (1:1). The experimental intervention will be Guizhi-Shaoyao-Zhimu decoction plus the conventional medicine methotrexate and the control intervention will be placebo plus the conventional drug methotrexate for 3 months. In addition, both groups will receive folic acid during treatment to prevent side effects from methotrexate. The primary outcomes are the erythrocyte sedimentation rate (ESR), C-reactive protein (CRP), hepatic and renal function, visual analog scale, disease activity score in 28 joints, measurement scale for TCM symptoms, and 7-joint ultrasound score.

**Discussion:**

We designed this double-blinded randomized controlled clinical trial to evaluate the efficacy and safety of Guizhi-Shaoyao-Zhimu decoction in RA patients using MSUS. The results of this trial may provide insights into how to improve the clinical symptoms of RA patients and delay further joint destruction. We hope that this trial may provide preliminary evidence of the efficacy of Guizhi-Shaoyao-Zhimu decoction in treating RA patients and that these results aid researchers, practitioners, and patients alike.

**Aim:**

The main aim of the study is to clarify the efficacy and safety of Guizhi-Shaoyao-Zhimu decoction in patients with early RA.

**Trial registration:**

Chinese Clinical Trials Register ChiCTR2000036141. Registered on 21 August 2020 (retroactively registered)

## Background

Rheumatoid arthritis (RA) is a chronic inflammatory autoimmune disease with the primary clinical symptoms of joint swelling and pain. Patients with severe conditions will suffer from bone destruction, joint deformities, and movement disorders. Globally, the incidence of RA is about approximately 0.8%, and the patient’s health and quality of life are greatly affected [1]. The main purpose of RA treatment is to reduce joint pain, prevent further deformation of the joints, and improve patients’ quality of life [2]. When treating RA at an early stage, optimal efficacy can usually be achieved. During this period, treatment can offer long-term benefits that can permanently change the outcome of the disease [3]. Early detection of erosion and synovial inflammation at an active stage resulting from RA can prevent damage to the joints and activity restrictions. On the other hand, it is very important to evaluate whether RA is in an active stage to provide appropriate treatment options, to evaluate of efficacy, and to aid in prognosis. Currently, Western medicine treatments for RA are mainly NSAIDS, glucocorticoids, DMARDS, and biological agents. However, many patients do not respond to these treatments, and thus, the development of newer, safer drugs is urgently needed. The advantages of treating RA with TCM have been gradually revealed, including in the form of the overall management of patients, fewer side effects, obvious efficacy, effective disease control, and improvement in the quality of life of patients. Thus, TCM can fill the gap in Western medicine.

Guizhi-Shaoyao-Zhimu decoction, which was originally from the book “*Synopsis of Golden Chamber*,” is a commonly used prescription. It is mainly composed of ginger, raw ephedra, prepared licorice, fangfeng, white peony root, cassia twig, aconite root, and common anemarrhena rhizome. It has the functions of promoting blood circulation and relieving pain, providing symptom relief for RA patients. Research has shown that compared to Western medicine, Guizhi-Shaoyao-Zhimu decoction has equal or higher efficacy and safety [4]. Other research [5] has shown that Guizhi-Shaoyao-Zhimu decoction has an anti-rheumatic effect on CIA rats. Its possible mechanism may be related to the inhibition of inflammation, suppression of the invasion and migration of synovioblasts, and induction of apoptosis in synovioblasts.

With the widespread use of musculoskeletal ultrasound (MSUS), it holds great value for evaluating of the degree of joint damage in RA patients, guiding the clinical selection of treatments, and assessing of prognosis. Research [6] has shown that MSUS can be used to measure and identify early cartilage lesions in RA patients in both the short and long term. In addition, due to advances in radiology, it has been reported that [7] the synovial blood flow signal cumulative correlation count of an early-stage RA patient has a predictive effect. Synovial blood flow signal accumulation in the joint, RF level, and laboratory indicators of inflammation are regarded as predictors of disease progression.

Therefore, we designed a double-blinded randomized controlled clinical trial to measure the safety and efficacy of Guizhi-Shaoyao-Zhimu decoction in treating early-stage RA.

## Methods

### Objective

The main objectives of this study are as follows: (1) to evaluate the changes of disease activity and joint ultrasound in patients with early RA treated with Guizhi-Shaoyao-Zhimu decoction and (2) to clarify the efficacy and safety of Guizhi-Shaoyao-Zhimu decoction in patients with early RA.

### Sample size calculation

To determine the sample size, we inquired about the previous research contents. The effective rate of methotrexate in the treatment of rheumatoid arthritis was 40% [8], and that of Guizhi-Shaoyao-Zhimu decoction was 64% [9]. If *α* = 0.05 and the test efficacy 1 − *β* = 0.80, then the sample size; thus, *n* = 122, with 61 patients in each treatment group. Considering a 20% dropout rate, the planned sample size for randomization was increased to 152.

$$ {n}_1={n}_2=\frac{{\left[{Z}_{\alpha /2}\sqrt{2p\left(1-p\right)}+{Z}_{\beta}\sqrt{p_1\left(1-{p}_1\right)+{p}_2\left(1-{p}_2\right)}\right]}^2}{{\left({p}_1-{p}_2\right)}^2} $$

### Trial design and setting

**Fig. 1:**
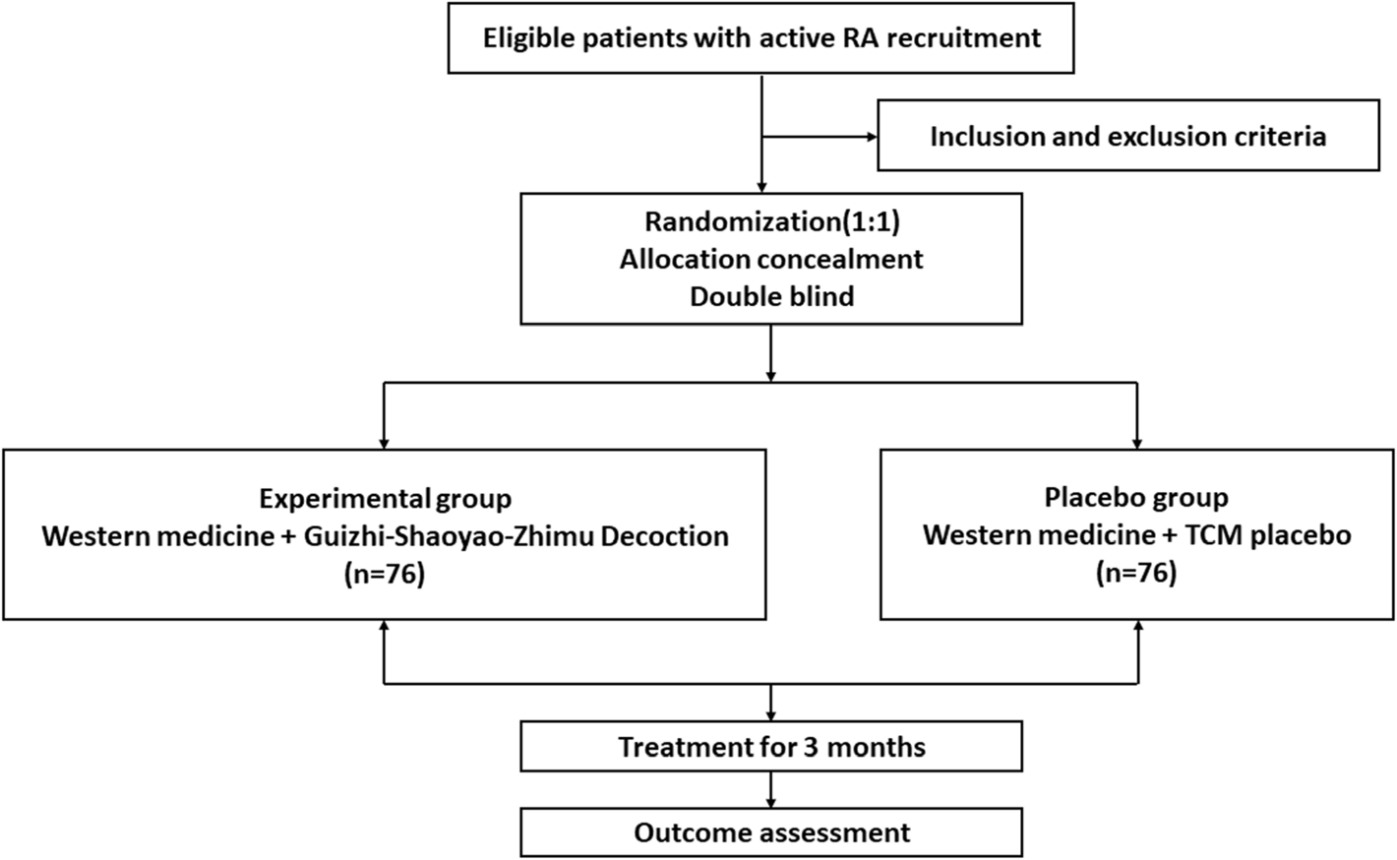
Study flow chart

This study is a randomized, double-blinded, parallel group, placebo-controlled trial. Eligible participants will be randomly assigned to the experimental group (Guizhi-Shaoyao-Zhimu decoction) or the control group with a 1:1 ratio. The subjects will be selected from patients with early active rheumatoid arthritis who visited the Rheumatology Department of Shuguang Hospital affiliated with Shanghai University of Traditional Chinese Medicine. We plan to recruit patients from participating hospitals via poster, networking, or WeChat. The posters will be placed on bulletin boards or other assigned places in the hospitals. At least one staff member (a postgraduate or doctor) will specialize in patient recruitment. Their contact information and clinic for screening visit will be detailed described in the recruitment advertisement. The design of the study follows a strict scientific clinical research methodology and complies with the principles outlined in the Declaration of Helsinki and the guidelines for good clinical practice. At any stage of the clinical study, a participant can withdraw from the clinical research without discrimination or retaliation. Patient rights and interests are not affected. If harm or injury related to the clinical research occurs, then the subjects can be appropriately compensated. The clinical study can be conducted only after the signature of the subject or legal representative/guardian is received with the date specified. If the subject or legal representative/guardian cannot read, a witness should be present. After a detailed explanation of the informed consent form, the subject or legal representative/guardian can provide consent, and the witness can sign and date the form. The flow chart and trial schedule are shown in Fig. 1 and Table 1, respectively.

**Table 1:** Schedule of study procedures

		Study period					
	Enrolment	Allocation	Post-allocation (days)				Close-out ***90d+***
**Time point**	***- -T0d***	***-T0d***	***T0d***	***T30d***	***T60d***	***T90d***	
**Enrolment**							
Patients with early RA	X						
RF	X						
Diagnosis and investigation of the distribution of TCM syndrome types	X						
**Informed consent**	X						
General information	X						
General physical examination	X						
**Allocation**		X					
**Interventions**							
Western medicine as well as TCM placebo combined group			X	X	X	X	
Western medicine as well as TCM combined group			X	X	X	X	
**Assessments**							
Blood routine			X	X	X	X	
Urine routines			X			X	
Hepatic and renal function (ALT, AST, AKP, eGFR, Scr, BUN)			X	X	X	X	
TCM symptoms			X	X	X	X	
ESR, CRP			X	X	X	X	
DAS28, VAS			X	X	X	X	
US7			X			X	
sCD14, MMP3, TIMP1			X			X	
Adverse events			X	X	X	X	

A total of 152 participants will be randomized into the control group or the experimental group. Assessors blinded to the randomization will collect data during the intervention period at baseline and at study months 1, 2, and 3. The peripheral blood levels of matrix metalloproteinase 3 (MMP3), matrix metalloprotease 1 (TIMP1), and soluble cluster of differentiation 14 (sCD14) will be detected before and after treatment to explore the biochemical mechanisms of Guizhi-Shaoyao-Zhimu decoction for the treatment of active RA

### Eligibility criteria

To participate in the trial, potential participants must fulfill all the following criteria:

#### Diagnostic criteria

1. Diagnostic criteria for RA: According to the criteria for the classification of RA proposed by the American College Rheumatology (ACR) and European League against Rheumatism (EUHLAR) in 2010, participants with a score of 6 or above who were diagnosed with RA [10].
2. Diagnostic criteria for active RA: Disease Activity Score (DAS28) > 2.6. DAS28, the most commonly used comprehensive score in RA, is used to evaluate the disease activity of RA patients with scores ranging from 0 to 10 [11]. According to EULAR diagnostic criteria for RA activity, DAS28 > 2.6. This project aims to study active RA patients, so the participants in this study should have a DAS28 score greater than 2.6.
3. Diagnostic criteria for TCM syndrome differentiation: according to the Guidelines for Clinical Research of Chinese Medicine (New Drug) [12], the participants had to present with at least two of the primary symptoms and more than two of the secondary symptoms listed in the following to be diagnosed with wind dampness heat bi syndrome.
  - Primary signs and symptoms:
    I Redness and swelling of joints
    II Acute joint pain
    III Stiff joints in the morning
    IV Activity limitation
  - Secondary symptoms:
    I Aversion to cold with fever
    II Thirsty upset
    III Dry stools and yellow urine
    IV Red tongue with yellow or dry fur
    V Rapid and slippery pulse

#### Inclusion criteria

Patients who meet the following criteria will be deemed eligible for the trial:

- Meet the diagnostic criteria for RA, meet the diagnostic criteria for active RA, and meet the wind dampness heat bi syndrome
- Aged 18–80 years with a disease course of less than 1 year
- Disease Activity Score in 28 joints-Erythrocyte Sedimentation Rate (DAS28-ESR) ≥ 2.6 (rounded to 1 decimal place)
- Patients receiving NSAIDs and hormone therapy were given a stable dose for at least 30 days before entering the trial and remained at the same dose for the rest of the treatment.
- Treatment with other DMARDs must be interrupted for at least 30 days before entering the trial.

#### Exclusion criteria

Patients who meet the following criteria will be excluded from the trial:

- Patients with interstitial lung disease accompanied by severe elevations of liver enzymes
- Women who are pregnant, breastfeeding, or who have recently prepared for childbirth
- Persons who are allergic or sensitized to the test drugs
- Patients with severe primary diseases of the heart, liver, kidneys, brain, endocrine system and hematopoietic system, psychiatric disorders, etc.
- The presence of other rheumatic diseases such as systemic lupus erythematosus, Sjogren’s syndrome, and myositis.

#### The drop out criteria

Patients will be terminated or withdrawn from the trial if they encounter any of the following conditions:

- Violation of any of the key inclusion or exclusion criteria
- Failure to take prescribed drugs during the trial, affecting the efficacy evaluation
- Taking other TCM that are prohibited by this protocol, so that the efficacy and safety of the treatment cannot be correctly judged
- Serious adverse reactions to the experimental or control drugs
- The desire to withdraw from the experiment
- Loss to follow-up, death, or withdrawal from the trial due to any other reason

### Interventions

#### TCM Guizhi-Shaoyao-Zhimu decoction preparations and placebo preparation

Traditional Chinese medicine decoctions are made by boiling herbs in boiling water for hours. However, for the convenience of administration, handling, distribution, and storage, Guizhi-Shaoyao-Zhimu decoction and placebo granules will be distributed to the participants. The preparation of Guizhi decoction and placebo will be entrusted to Jiangsu Tianyin Pharmaceutical Co, Ltd., and all operations will strictly follow the procedures stipulated in the Chinese National Pharmacopoeia. The placebo is composed of starch, dextrin, and bitters, and its smell and taste are similar to those of traditional Chinese medicine granules. The composition and function of all herbs in Guizhi decoction are summarized in Table 2.

**Table 2:** Composition and action of Guizhi-Shaoyao-Zhimu decoction in Chinese herbal medicine

Ingredient	Granule dose	Action (TCM)
*Cinnamomum cassia* (guizhi)	4.5 g	1. Relieving exterior syndrome by diaphoresis 2. Harmonizing yingfen and weifen 3. Warming the channel and activating blood circulation warming the meridian to relieve pain
Radix of *Paeonia lactiflora* (Shaoyao)	4.5 g	1. Nourishing the blood 2. Reducing yin 3. Soothing the liver 4. Relieving pain stabilizing yin and yang in the liver
Radix and Rhizoma of *Glycyrrhiza uralensis* (gancao)	3 g	1. Clearing heat and removing toxicity 2. Invigorating spleen and supplementing qi moderating the property of herbs
Rhizoma of *Anemarrhena asphodeloides* (Zhimu)	4.5 g	1. Clearing heat-fire nourishing yin for moistening dryness
Rhizoma of *Atractylodes macrocephala* (Baizhu)	4.5 g	1. Invigorating the spleen replenish qi
Rhizoma of *Zingiber officinale* (Shengjiang)	3 g	Resolve the exterior and dissipate cold
Radix of *Saposhnikovia divaricata* (Fangfeng)	4.5 g	1. Expelling wind to relieve superficies 2. Removing dampness to relieve pain
Processed lateralis Radix of *Aconitum carmichaeli* (Paofuzhi)	1.5 g	1. Tonifying fire and helping yang 2. Dispelling cold and removing dampness

#### Conventional Western medicine preparation

For methotrexate tablets, specification was as follows: 2.5 mg × 16 tablets/bottle; manufacturer: Shanghai Xinyi Pharmaceutical Co., Ltd.; national drug approval number H31020644.

For folic acid tablets, it was as follows: 5 mg × 100 tablets/bottle; manufacturer, Shanghai Xinyi Huanghe Pharmaceutical Co., Ltd.; national drug approval number H31020147.

#### Medication method

For the experimental group (Western medicine + Guizhi-Shaoyao-Zhimu decoction), Guizhi-Shaoyao-Zhimu decoction will be taken twice a day (30 g each time). Combined MTX will be taken orally once a week (10~15 mg). Folic acid tablets will be taken orally twice a week (5 mg).

For the control group (Western medicine + TCM placebo), TCM placebo twice a day, 30 g each time. MTX is taken orally 10~15 mg once a week. Folic acid tablets should be taken 5 mg orally twice a week.

Each patient lasted for 3 months.

#### Combination of drugs

For combination of drugs, according to ethical requirements, if the patient’s pain is unbearable and the doctor considers it necessary to add other drugs to the regimen, drug combinations can be used. The combination of drugs is limited to nonsteroidal anti-inflammatory drugs and low-dose hormones. The dosage and starting and ending time will be recorded in detail.

### Measurement items and time points of data collection

#### Outcome measure

##### General information (before treatment)

The patients’ general information, including demographic data, general medical examination items, and general clinical data will be collected at baseline.

- Demographic data: The patients’ name, sex, age, nationality, occupation ID number, date of birth, home address, and contact information will be collected.
- General medical examination items will include respiration, body temperature, pulse, blood pressure, tongue coating, and pulse measurements.
- General clinical data will include history of present illness, past medical history, family history, personal life history, menstrual history, marital history, allergy history, and social history.

##### Outcome variables

The primary outcome of this trial will be recorded at baseline and at weeks 1, 2, and 3.

1. Laboratory indexes including routine blood examination, ESR, CRP, and hepatic and renal function will be measured.
2. *The visual analog scale (VAS)* [13]. The pain VAS is self-completed by the respondent. Using a ruler, the score is determined by measuring the distance (mm) on the 10–cm line between the “no pain” anchor and the patient’s pain anchor, providing a range of scores from 0 to 100 [14, 15]. A higher score indicates greater pain intensity.
3. *Disease Activity Score in 28 joints-Erythrocyte Sedimentation Rate (DAS28-ESR)*. DAS28-ESR was calculated as 0.56 × √(Tender joint count) + 0.28 × √(Swollen joint count) + 0.7 × ln ESR + 0.014 × VAS-GH [16].
4. *Measurement scale for TCM symptoms*. To facilitate the evaluation of the results, we will use the measurement scale for TCM symptoms recommended by the Guidelines for Clinical Research of Chinese Medicine (New Drug) [12] to score the symptoms of the patients. The scale consists of 4 major symptoms and 7 minor symptoms. Each symptom is graded on 4 levels, ranging from zero, mild, medium, to severe, with corresponding scores of 0, 1, 2, and 3. The total score was calculated by adding up each score in the scale and using these scores to calculate an efficacy indicator (EI) for the evaluation of treatment efficacy.

EI = (Total symptom score at baseline − Total symptom score post-treatment)/Total symptom score at baseline × 100%

The degree of symptom improvement will be presented in four categories ranging from full recovery (EI ≥ 90%), good recovery (90% > EI ≥ 70%), modest recovery (70% > EI ≥ 30%) to no recovery (EI < 30%).

5. *Ultrasonic assessment*. The 7-joint ultrasound score (US7) [17] is used to determine the degree of synovitis, synovial hyperplasia, and bone erosion. This score examines 7 joints (wrist, MCP2, MCP3, PIP2, PIP3, MTP2, and MTP5 joints) of the clinically dominant hand and foot of the patients. The synovitis, tenosynovitis, and bone erosion of each joint will be observed, with the highest score being 94 points according to the following standards.
  - Synovial thickening is graded on four grades according to no, light, medium, and heavy, and the corresponding scores are 0, 1, 2, and 3.
    I *0 points*. No synovial thickening
    II *1 point*. Mild synovial thickening (the thickened synovium is located only in the circumferential triangle of the bone)
    III *2 point*. Moderate synovial thickening (thickened synovial membrane extending from the circumferential triangle to the middle but not to the epiphysis)
    IV *3 points*. Severe synovial thickening (thickened synovial membrane extending from the circumferential triangle to the middle and metaphysis)
  - Synovitis of tenosynovitis is graded on two grades according to no or have, and the corresponding score is 0 and 1.
    I *0 points*. No hypoechoic thickened tissue with fluid and Doppler flow signal in the tendon sheath
    II *1 point*. Hypoechoic thickened tissue with fluid and Doppler flow signal in the tendon sheath
  - Bone erosion scored 0 or 1 on a no or yes basis.
    I *0 points*. Continuous absence of cortical bone defects
    II *1 point*. Cortical discontinuity in the joint cavity in two vertical planes
  - Synovitis. If synovial thickening is present, a color Doppler flow signal (PDUS) is additionally observed with color Doppler. The pulse repetition frequency (PRF) is set to a minimum of 0.7 to 1.0 KHz. The Doppler flow signal is graded on 3 levels.
    I *0 points*. No blood flow signal
    II *1 point (mild)*. A small amount of pitting blood flow signal.
    III *2 points (moderate)*. A continuous blood flow signal covering less than 50% of the joint cavity.
    IV *3 points (severe)*. The area of blood flow signal exceeds 50% of the joint cavity

### Safety guarantee and evaluation

All trial interventions will be conducted under the guidance of rheumatology experts. The researchers will closely observe the physical conditions of the participants. Routine hematuria and liver and kidney function tests will be performed before enrollment and at 1, 2, and 3 months after enrollment. Adverse events and serious adverse events will be recorded by all participants and will be reported to the principal investigator and professional leader in the event of adverse events to find a reasonable solution. Conduct monthly internal team review of adverse events related to drug use. In addition, the safety and efficacy of the intervention will be examined by two clinicians and statisticians independent of the trial, who will also make professional recommendations on the pace of trial recruitment, whether to continue and terminate the trial, and, if necessary, on amendments to the protocol. Subjects have the right to withdraw their consent to participate in the study at any time for any reason, without any consequences for further treatment.

### Sequence generation and allocation concealment mechanism

Use the online central random system of the Chinese Academy of Chinese Medical Sciences. The drug will be randomly coded according to the clinical trial randomization protocol and used as the subject’s unique identification code. Subjects are included in a certain order, and random numbers can be obtained online. Subjects are randomly assigned to the experimental group and the placebo group in a 1:1 ratio. A double-blind and placebo-controlled trial design is adopted. The patients are randomly assigned drug A or B, respectively, and sequenced according to maintain blinding. Subjects and researchers are blinded, and drug A and drug B are distributed in the order in which patients visit the clinic. If serious adverse events occur during the test, the emergency envelope should be unsealed immediately and delivered to the researcher of the research unit for storage. A double unblinding method will be used. At the end of the study, after verification of blinding, the data will be locked, and the statistical specialist who maintains the blinding will unblind the first level. Statistical experts will be informed of A/B group assignments corresponding to each case number for statistical analysis of all the data. When the statistical analysis and the summary report are completed, level II unblinding will be conducted at the clinical summary meeting to reveal the subjects in group A and group B.

### Data quality control

A training will be done for all participating staff on the trial protocol, usage of the randomization, data management systems, etc. The principle investigator will supervise the proceed of the trial at least once every month, collecting, assessing, reporting, and managing solicited and spontaneously reported adverse events and other unintended effects of trial interventions or trial conduct. An ethics committee will review conduct especially on safety, rights, and well-being of the participants at the middle and the end of the trial. The auditing will be done by Clinical Evaluation Center of the China Academy of Chinese Medical Sciences at the beginning, middle, and end of the trial.

### Data collection and management

Composition of data monitoring committee (DMC) is composed of professional statisticians. They will perform statistical analysis, participating in the entire process from the research design and implementation to the analysis and summary. A statistical analysis plan will be developed after the completion of the study program and completion of the case reporting forms, and the statistical analysis reports will be provided after the necessary modification of the data analysis is performed as necessary during the research process. According to the project of the case report form, EpiData3.1a software will be used to establish the corresponding entry procedure and set the logical examination qualifications at the time of entry, and the database will be piloted to establish a database system dedicated to this experiment. The signed case report form and the audit statement will be given to the data administrator, who will examine the date, group criteria, culling criteria, shedding criteria, and missing values. If there is doubt about “data question form,” it will be returned to the monitor, and the researcher will answer and sign the question in writing and return it to the data administrator; “data question form” should be properly stored to protect confidentiality before, during, and after the trial. The data are entered synchronously by the data administrator using a two-person entry method. The database will be checked for each item using the verification function in the EpiData3.1a software; any inconsistent result values will be reported. The original case reporting tables will be checked item by item, and 10 case reporting tables and the data in the database will be randomly selected for manual comparison to ensure that the data in the database are consistent with the results in the case reporting tables. The original CRFs and any other records will be archived for 5 years.

### Statistical analysis

An independent statistician will perform statistical analysis according to the statistical analysis plan using SPSS software (version 26; SPSS Inc., Chicago, IL, USA). Continuous variables will be expressed as the mean (standard deviation) if the data are normally distributed; otherwise, they will be expressed as the median (interquartile). Categorical variables will be presented as frequencies (percentages). Baseline analyses will be performed using analysis of variance or nonparametric tests for continuous variables, and chi-squared or Fisher’s exact tests for categorical variables, respectively. Changes from baseline to post-treatment in DAS28, VAS, TCM syndrome scores, and US7 will be analyzed using analysis of covariance with associated 95% confidence intervals. A two-sided *P* value of 0.05 or less is considered statistically significant. Multiple imputation will be used to handle any missing data.

### Monitoring and audit

An inspector appointed by the sponsor will conduct irregular monitoring visits on site or remotely. At any stage of the trial, auditors can check compliance with the protocol to ensure that no violations of basic principles of clinical practice have occurred. In addition, the inspector needs to check the accuracy, consistency, and completeness of the test data.

### Trial protocol amendments

The protocol may need to be modified depending on the progress of the trial. If any modification is required, all researchers will work with clinical and statistical experts to fully demonstrate the need for improvement before making any modification.

### Ancillary and post-trial care

This study was sponsored by Shanghai Municipal Science and Technology Commission, and the fund of the experiment was allocated by the organization. The organization manages the middle and final stages of the trial.

### Dissemination

The study results will be disseminated in Open Access and peer-reviewed journals and shared through oral and poster presentations at international conferences. All resources will be uploaded to an online knowledge management platform, and the research team will report their individual results to study participants.

## Discussion

According to the clinical manifestations of RA, it is included in the category of arthralgia disease in TCM. During the early stage of the disease, wind chill invades, blood vessels are blocked, Qi-blood transportation in the joints is affected, and veins are blocked, resulting in qi deficiency and blood stasis. As time passes, qi and the blood gradually become weak, becoming unable to nourish muscles and bones and causing local pain; notably, pain is the most difficult of these symptoms to relieve. Later, doctors began to use Guizhi-Shaoyao-Zhimu decoction to treat the disease, and the effect was considerable. In this trial, Chinese herbal granules were used instead of TCM decoction pieces to ensure the quality requirements of Chinese medicine ingredients in terms of drug concentration and efficacy.

The potential mechanism of Guizhi-Shaoyao-Zhimu decoction in the treatment of RA remains to be elucidated. Recent clinical research has shown that the clinical cure rate of Guizhi-Shaoyao-Zhimu decoction for RA patients may be between 87.5 and 95.8%, which is higher than that of indomethacin, tripterygium glycosides, and prednisone [18–20]. In addition to its significant efficacy, Guizhi-Shaoyao-Zhimu decoction has been used in clinical practice for many years, and no obvious adverse reactions have been reported [21]. Studies have shown that [22, 23] Guizhi-Shaoyao-Zhimu decoction can alleviate the progression of RA by inhibiting the differentiation and activities of osteoclasts, reducing the proliferation of synovial cells, and increasing the apoptosis of synovial cells both in vitro and in vivo.

MSUS can reveal the pathological changes that occur during early-stage RA and can be used to assess the activity level of RA patients. MSUS can help guide clinical treatment and has considerable advantages. Therefore, we used MSUS as an important monitoring method for the diagnosis and treatment of RA. After all, MSUS is an inspection method with the advantages of nonionizing radiation, safety, noninvasiveness, and ease of operation.

Based on the current evidence, we designed this double-blinded randomized controlled clinical trial. MSUS is used to evaluate the efficacy and safety treatment with Guizhi-Shaoyao-Zhimu decoction in RA patients. This trial complies with the SPIRIT 2013 [24] and SPIRIT 2013 statement, explanation, and elaboration [25], which covers scientific, ethical, and safety issues (Annex 1). The results of this trial may provide insights into how to improve the clinical symptoms of RA patients. The results of this trial may help to improve their clinical symptoms and to delay further joint destruction. We hope that this trial may provide preliminary evidence for the efficacy of Guizhi-Shaoyao-Zhimu decoction in treating RA patients and that these results aid researchers, practitioners, and patients alike.

There are still some limitations in this study. First, MSUS cannot fully assess the condition of the joint because of its poor of penetration ability. Moreover, MSUS is the imaging examination that is most subjectively affected by the operator. The quality of the ultrasound image depends on the examiner’s experience, the resolution of the instrument, and the placement of the ultrasound probe. On the other hand, there is currently no international uniform standard for MSUS in evaluating RA patients. Second, the study will be conducted in Shanghai, China. It is not certain whether the relative effects of Guizhi-Shaoyao-Zhimu decoction will be similar in other ethnic groups. Third, which components in Guizhi-Shaoyao-Zhimu decoction have an effect on the therapeutic effect requires further research and exploration.

## Data Availability

Does not apply.

## Abbreviations

RA: Rheumatoid arthritis
MSUS: Musculoskeletal ultrasound
ESR: Erythrocyte sedimentation rate
CRP: C-reactive protein
MMP3: Matrix metalloproteinase 3
TIMP1: Matrix metalloprotease 1
sCD14: Soluble cluster of differentiation 14
ACR: American College Rheumatology
EULAR: European League against Rheumatism
DAS28: Disease activity score
VAS: Visual analog scale
PRF: Pulse repetition frequency
